# Severe acute hypoxia upregulates anaerobic metabolism in non-reproductive but not queen naked mole-rats

**DOI:** 10.1242/jeb.250397

**Published:** 2025-07-28

**Authors:** Mohammad Ojaghi, Matthew E. Pamenter

**Affiliations:** ^1^ Department of Biology, University of Ottawa, Ottawa, ON, Canada, K1N 9A4; ^2^ Ottawa Institute of Systems Biology, Ottawa, ON, Canada, K1H 8M5

**Keywords:** Hypoxic metabolic response, Development, Juvenile, Breeder, Glycolysis

## Abstract

Most vertebrates upregulate anaerobic metabolism in severe hypoxia, which results in metabolic acidosis that must be resolved during reoxygenation. Naked mole-rats (NMRs) are hypoxia-tolerant mammals and drastically reduce their metabolic rate while maintaining systemic pH homeostasis during acute hypoxia. Whether NMRs employ anaerobic metabolism in hypoxia is currently debated. Given the robust systemic hypoxic hypometabolism of this species, we hypothesized that anaerobic metabolism is recruited on a tissue-specific basis that varies between developmental stages and colony caste position. To test this, we treated subordinate juvenile and adult, and breeding (queen) NMRs in normoxia (21% O_2_) or hypoxia (3% O_2_) for 1 h, and then measured blood lactate, glycolytic enzyme activity, and the expression of genes that encode for enzymes involved in glycogen and glucose metabolism, and lactate transport. We found that (1) blood lactate levels increase similarly during hypoxia across developmental stages and castes, but that (2) glycolytic activity increased or remained stable in subordinates and juveniles but was unchanged or reduced in queens; (3) MCT4 gene expression decreased markedly in subordinate and juvenile brain and increased in muscle and kidney, but was unchanged in queens; and (4) the expression of genes associated with glycogenolysis and gluconeogenesis varied across tissues in subordinates/juveniles with some markers being down or upregulated or unchanged, but were always unchanged or downregulated queens. Taken together, our results suggest that hypoxia upregulates glycolysis and glycogen mobilization in subordinates and juveniles, but not in queens.

## Teaser

**Summary:** Anaerobic metabolism is differently upregulated between hypoxic queen and subordinate naked mole-rats.

## INTRODUCTION

Hypoxia curtails aerobic metabolic pathways (Pamenter, 2014). As a result, when most animals encounter hypoxic environments, they are unable to balance metabolic oxygen (O_2_) demands to match the reduced O_2_ content in the inhaled air (Ayalew et al., 2021; Childress and Seibel, 1998; Dzal et al., 2015; Jensen, 2009; Seibel and Deutsch, 2020). Instead, most mammals rely on anaerobic metabolism to meet energy demands when aerobic energy provisions are compromised by environmental hypoxia (Buck and Pamenter, 2006; Hochachka, 1986). Anaerobic metabolism, primarily via the glycolytic pathway, leads to accumulation of lactate and acidification of the cellular milieu. Monocarboxylate transporter 4 (MCT4) plays a critical role in lactate export from cells, particularly in tissues where glycolysis predominates under low O_2_ availability (Contreras-Baeza et al., 2019). This transporter thus helps to maintain cellular pH homeostasis by facilitating lactate clearance during and following hypoxia. Upon reoxygenation, lactate is oxidized, converted to glucose or glycogen, or used in protein synthesis (Warren and Jackson, 2008).

Many animals are resident to hypoxic niches and have evolved adaptations to cope with the curtailment of aerobic metabolism and accumulation of anaerobic end-products during periods of hypoxia (Bickler and Buck, 2007; Dzal et al., 2015; Houlahan et al., 2018; Kirby et al., 2018; Ojaghi and Pamenter, 2024; Seebacher, 2009; Storz and Scott, 2019; Zhang et al., 2023). Among these champions of hypoxia tolerance are naked mole-rats (NMRs; *Heterocephalus glaber*), which are one of the most hypoxia-tolerant mammals and are capable of significantly lowering their metabolic rate to reduce energy demands during periods of hypoxia exposure (Buffenstein et al., 2022; Edrey et al., 2011; Pamenter, 2022). For example, in severe hypoxia (3% O_2_), NMRs reduce their metabolic rate by >85% while remaining active and alert (Houlahan et al., 2018; Ilacqua et al., 2017; Kirby et al., 2018), unlike other hypoxia-tolerant animals that typically enter a torpor-like state when O_2_ availability is limited (Guppy and Withers, 1999; Hayden and Lindberg, 1970). Remarkably, NMRs achieve this degree of metabolic rate suppression without developing significant metabolic acidosis or O_2_ debt (Pamenter et al., 2019). This resilience may be due to their unique capacity to switch metabolic substrates, to enhance the efficiency of energy production and/or to utilize buffering mechanisms that mask typical signs of metabolic acidosis (Pamenter, 2022, 2023).

Mitochondrial and proteomic studies in various NMR organs suggest that subordinate (non-breeding adult) NMRs may employ a tissue-specific suite of metabolic responses to hypoxia. For example, in brain there is significant metabolic suppression (Farhat et al., 2020, 2021b; Pamenter et al., 2018), with a shift towards reliance on the pentose phosphate pathway to maintain redox balance and produce NADPH, which also protects against oxidative stress (Cheng et al., 2022). In brown adipose tissue, non-shivering thermogenesis ceases, helping NMRs lower their body temperature to conserve energy (Cheng et al., 2021b). In skeletal muscle, mitochondrial function is only mildly reduced by hypoxia (Cheng et al., 2021a), but AMPK (5′-AMP-activated protein kinase) activity is downregulated (Hadj-Moussa et al., 2021). This downregulation, associated with decreased expression of key AMPK subunits such as AMPK-α2 and phosphorylated AMPK (p-AMPK), leads to reduced expression of proteins involved in glucose metabolism, such as glucose transporter 4 (GLUT4) and phosphorylated glycogen synthase, consistent with reduced metabolic flux through skeletal muscle. Conversely, cardiac muscle may increase metabolic demands in hypoxia and has large glycogen stores, which are crucial during hypoxic conditions. These glycogen reserves can be utilized to produce ATP, ensuring that the heart continues to function effectively even in hypoxia (Faulkes et al., 2019). Indeed, the NMR heart maintains or even increases its metabolic activity during hypoxia relative to systemic energy demands by sustaining the activity of key enzymes involved in glycolysis, the tricarboxylic acid (TCA) cycle and fatty acid oxidation (Farhat et al., 2020).

At the whole-organismal level, NMR blood glucose concentrations increase during severe hypoxia and systemic fuel substrate use shifts from lipids to carbohydrates (Ojaghi and Pamenter, 2024; Pamenter et al., 2019), possibly because of a net reduction of glucose uptake by tissues and/or increased glucose release into the bloodstream. Indeed, glucose homeostasis in the NMR liver is altered in hypoxia, possibly conserving energy by reducing glucose release in moderate hypoxia (Cheng et al., 2022), but then increasing glucose release to the blood during severe hypoxia, as indicated by a decrease in liver glucose levels alongside a concurrent increase in blood glucose at only very severe levels of hypoxia exposure (<∼3% O_2_) (Ojaghi and Pamenter, 2024; Pamenter et al., 2019).

Taken together, these studies suggest that NMRs recruit a wide range of tissue-specific strategies depending upon the severity of environmental hypoxia. It is thus likely that different tissues use aerobic and anaerobic metabolic pathways to varying degrees, depending on the hypoxic stimulus, but this has not been comprehensively explored. Furthermore, almost all that is known about the NMR response to hypoxia comes from studies in subordinate adult animals, and little to nothing is known about how juvenile or reproductive animals respond to hypoxia. This is an important consideration as NMR colonies are divided into various castes, with subordinate animals doing most of the colony work (e.g. foraging and burrowing) and reproductive animals being less physically active and spending more time in the nest (Brett, 1991; Buffenstein et al., 2022). To address this, we asked to what degree anaerobic metabolic pathways are activated during severe hypoxia in several major tissues and across developmental stages in NMRs.

Specifically, and building upon our knowledge that robust metabolic rate suppression in hypoxic NMRs reduces their reliance on anaerobic pathways by minimizing overall energy demands, we hypothesized that upregulation of anaerobic metabolism would occur in a selective and tissue-specific manner in this species, and that this recruitment would vary by developmental stage and/or caste status. We predicted that (1) glycolytic enzyme activity would vary by tissue such that activity in tissues with high obligatory metabolic activity (such as brain, heart and liver), increases to meet neuronal and cardiac energy demands, and to mobilize glucose for systemic energetic support, respectively; conversely, we predicted that glycolytic enzyme activity in muscle and kidney would decrease to conserve energy in these less critical tissues. We also predicted that (2) expression of genes encoding glycogen metabolism and gluconeogenesis enzymes, which are O_2_-dependent processes, would be downregulated in non-essential tissues such as muscle and kidney to conserve energy, but maintained or upregulated in essential tissues such as liver to support systemic glucose production; and (3) MCT4 expression would be downregulated, which may impair lactate export from tissues. Considering developmental stages and caste, we predicted that (4) juvenile and subordinate NMRs would exhibit greater engagement of anaerobic metabolic pathways under hypoxia compared with queens, consistent with their more active roles in the colony as workers requiring more energy to perform physical tasks, whereas queens who spend more time resting, may show lower activation of these pathways. To test these predictions, we exposed juvenile (8 weeks old), adult subordinate (1–13 years old) and queen (3–12 years old) NMRs to 1 h of normoxia (21% O_2_) or hypoxia (3% O_2_), and measured blood lactate concentration, glycolytic enzyme activity, and the expression of enzymes involved in gluconeogenesis, glycogen synthesis and glycogenolysis, and lactate transport.

List of abbreviations

*Actb*: *β-actin*
AMPK: AMP-activated protein kinase
ATP: adenosine triphosphate
CO_2_: carbon dioxide
*EEF2*: *eukaryotic elongation factor 2*
G6Pase: glucose 6 phosphatase
GYS2: glycogen synthase
LDH: lactate dehydrogenase
*MCT4*: *monocarboxylate transporter 4*
NADPH: nicotinamide adenine dinucleotide phosphate
NMR: naked mole-rat
O_2_: oxygen
PCK: phosphoenolpyruvate carboxykinase
PHKG1/2: phosphorylase kinase gamma 1/2
PK: pyruvate kinase
PYG: glycogen phosphorylase

## MATERIALS AND METHODS

### Animals and ethics

A total of 24 naked mole-rats (*Heterocephalus glaber* Rüppell 1842) were included in this study, including eight adult subordinates (1–13 years old; 55±10 g), eight queens with previous successful litters but not currently pregnant or lactating (3–12 years old; 65±8 g), and eight juveniles (∼2 months old; 13±2 g). The animals were housed in interconnected multicage systems with controlled environmental conditions (30°C; 70% humidity; 12 h:12 h light:dark cycle) and were provided with fresh tubers, vegetables, fruits and Pronutro cereal supplement *ad libitum*. Animals were not fasted prior to treatment and experiments were conducted at the same time of day to avoid confounding effects of circadian rhythms. All experimental protocols were approved by the University of Ottawa's Animal Care Committee in accordance with the Animals for Research Act and the guidelines of the Canadian Council on Animal Care.

### Experimental design

Animals were equally and randomly divided between a normoxic control group and a hypoxic treatment group. Animals were placed in a chamber with continuous airflow composed of either normoxic (21% O_2_, 0.04% CO_2_, balance N_2_) or hypoxic (3% O_2_, 0.04% CO_2_; balance N_2_) gas mixtures for 1 h. After exposure, animals were quickly killed by cervical dislocation, followed by immediate decapitation. Animals were handled gently to minimize stress prior to decapitation as stress-induced muscle contraction could contribute to lactate production, and were decapitated within 10 s of removal from the treatment chamber. Anaesthesia was not used to avoid introducing confounding effects on blood and tissue lactate levels. Blood was collected for lactate measurements and tissues, including the brain, heart, liver, muscles and kidney, were sampled within 2 min over ice, immediately frozen in liquid nitrogen, and stored at −80°C until molecular analysis.

### Blood lactate

Approximately 30 μl of blood was used to measure lactate levels with a StatStrip Xpress Lactate Analyzer (Nova Biomedical, Waltham, MA, USA).

### Measurement of gene expression by real-time RT-PCR

Total RNA was extracted from 50–100 mg of frozen, powdered tissue using TRIzol LS reagent (Thermo Fisher Scientific) according to the manufacturer's instructions. RNA concentrations and purities were determined using a NanoDrop ND-1000 Spectrophotometer, and the RNA was stored at −80°C. Following genomic DNA removal, cDNA was synthesized using the iScript™ cDNA Synthesis Kit (Bio-Rad) according to the manufacturer's instructions. The cDNA was then stored at −20°C.

Real-time reverse transcription (RT)-PCR was conducted using Maxima SYBR Green qPCR Master Mix (Bio-Rad) on a Rotor-Gene Q real-time PCR machine (Qiagen). Reactions were performed in a total volume of 10 µl according to the manufacturer's instructions. Primer sequences used in this study are listed in Table 1. Two reference genes, *eukaryotic elongation factor 2* (*EEF2*) and *β-actin* (*Actb*), were used in this study. These genes were selected based on their stable expression across all experimental conditions and tissues, as validated in a previous study (Nguyen et al., 2019) and confirmed in our study (data not shown). Each sample was run in duplicate, accompanied by negative controls, including a no-template control (where cDNA was replaced with water) and a no-RT control (where the cDNA synthesis reaction was carried out without reverse transcriptase). The cycling conditions were as follows: 30 s at 95°C, followed by 40 cycles of 10 s at 95°C and 25 s at 60°C. Melt curve analysis was conducted as per the manufacturer's protocol. Standard curves were generated using serially diluted pooled samples, and relative transcript abundances were calculated using the Pfaffl method (Pfaffl, 2001).

**Table 1.** Primer sequences used for qPCR in this study

Gene	Forward primer (5′ to 3′)	Reverse primer (5′ to 3′)
*MCT4*	CTGTGGATGTGAGGGTGGAC	CTCCCCGTTTTTCTCAGGCT
*PHKG1*	CTGCTCAGGGGACATGGTTT	GGGGGTGTCTGATCTCTTGC
*PYGL*	GATTGGCTCAGGCATGGAAAC	ACGGGGGTGTCATAAGGGA
*GYS2*	GGACGCCATGAATAAGCACG	CAGGTTCCAGGCAGAGTAGC
*PHKG2*	TTTTGGGTTCTCCTGCCACTT	CATAGCCTGGGTGGGTTTCAT
*PYGM*	ACTCATCACTGCCATTGGGG	ATCACCTTTTCAGCCAGCGA
*PYGB*	GAACCTGTGACACTCTCCTGG	AGGCATCTCTGCTTTCTGTCC
*PCK*	CAGCTCACACCCATTGGCTA	TACGTTGACATTCCCCAGGC
*G6pase*	TGTCAGAAGTTGCGTCCTCC	AGCACACCTGGTGAAGTCTC
*Eukaryotic elongation factor 2*	CTGCCAGCTCATCCTAGACC	CTTGTCCTTGTCCTCGCTGT
*Actb*	CTCTGTGTGGATCGGTGGC	GGGTGAAAGGCAGCGAAGTA

### Enzyme assays

Enzyme activities were determined using a Spectra Max Plus384 Absorbance Microplate Reader (Molecular Devices, Sunnyvale, CA, USA). To assess the activities of the key glycolytic enzymes pyruvate kinase (PK) and lactate dehydrogenase (LDH), 50 mg of frozen tissue samples were weighed and homogenized on ice in 19 volumes of extraction buffer [25 mmol l^−1^ Tris·HCl, 1 mmol l^−1^ EDTA, with 5 mmol l^−1^ dithiothreitol (DTT) and 0.05% (vol/vol) Triton X-100 added fresh on the day of the experiment]. The homogenates were centrifuged at 2400 ***g*** for 5 min at 4°C, and the supernatant was stored at −80°C until further analysis. Assay conditions were optimized using skeletal muscle to achieve maximal enzyme activity, which may not represent the maximal rate in all tissue types. Homogenates underwent a freeze/thaw cycle, and preliminary tests confirmed that substrate and cofactor concentrations were saturating but not inhibitory. Control reactions, lacking substrate, were run in parallel for each enzyme to account for any background activity. All assays were conducted in triplicate at the NMR body temperature of 32°C under the following conditions (Farhat et al., 2020): PK [A340; pH 7.35] (Zammit et al., 1978): 0.17 mmol l^−1^ NADH, 5 mmol l^−1^ ADP, 80 mmol l^−1^ KCl, 10 mmol l^−1^ MgCl_2_ and 5 mmol l^−1^ phosphoenolpyruvate (PEP) (omitted from the control), with excess coupling enzyme (LDH) in 160 mmol l^−1^ triethanolamine/HCl; and LDH [A340; pH 7.3] (Zammit and Newsholme, 1976): 0.17 mmol l^−1^ NADH, 1 mmol l^−1^ KCN and 2 mmol l^−1^ pyruvate (omitted from the control) in 50 mmol l^−1^ Tris·HCl.

### Statistical analysis

All statistical analyses were performed using GraphPad Prism software (GraphPad Software, San Diego, CA, USA). Significant differences (*P*<0.05) were determined using one-way and two-way ANOVA to assess intraspecific differences between normoxia and hypoxia for each independent variable. When a significant interaction or main effect was identified, Tukey's multiple comparisons test was applied to determine specific differences between normoxia and hypoxia within each treatment group. Data are expressed as means±s.e.m., with asterisks or letters marking significant differences as indicated in the figure legends.

## RESULTS

### Severe acute hypoxia increases blood lactate in all developmental stages

**Fig. 1.**
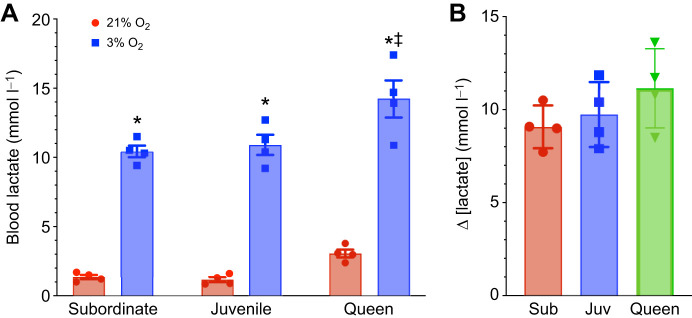
**Acute hypoxia elevates blood lactate in all naked mole-rat castes, with a similar magnitude of increase across groups.** (A) Acute hypoxia increases blood lactate across developmental stages in naked mole-rates (NMRs; *n*=4 for each group). Animals were exposed to 21% (red bars) or 3% O_2_ (blue bars) for 1 h. (B) Change in blood lactate levels between normoxia and hypoxia for each group. Data are presented as means±s.e.m. Asterisks (*) indicate significant effects of hypoxia within each caste. Double daggers (‡) indicate significant differences between queens and subordinate animals (one- and two-way ANOVA, *P*<0.05).

Analysis with a two-way ANOVA revealed a significant effect of hypoxia on blood lactate concentration across all developmental stages, but no differences in normoxic lactate concentration or the magnitude of hypoxia-mediated increases in lactate across developmental stages (effect of hypoxia on blood lactate; *n*=4 for each group and treatment, *F*_1,18_=337.8, *P*<0.0001; Fig. 1A). Specifically, in normoxia, subordinates and juveniles exhibited relatively low blood lactate levels (∼1.2 mmol l^−1^), whereas queens had ∼2-fold more lactate (∼3 mmol l^−1^); however, this difference between groups was not significant (*P*=0.3635 versus juveniles and 0.4710 versus subordinates). As a result, the maximum blood lactate concentration in queens during hypoxia was ∼30% higher than that of the other developmental stages (*F*_2,18_=10.92, *P*=0.0008 for the effect of developmental stage groups on blood lactate). However, analysis of the magnitude of change across ontogeny indicated that there was no difference in the net increase of blood lactate between groups (*F*_2,9_=1.524, *P*=0.2691; Fig. 1B).

### Hypoxia alters *MCT4* expression differently between breeding and non-breeding NMRs

**Fig. 2.**
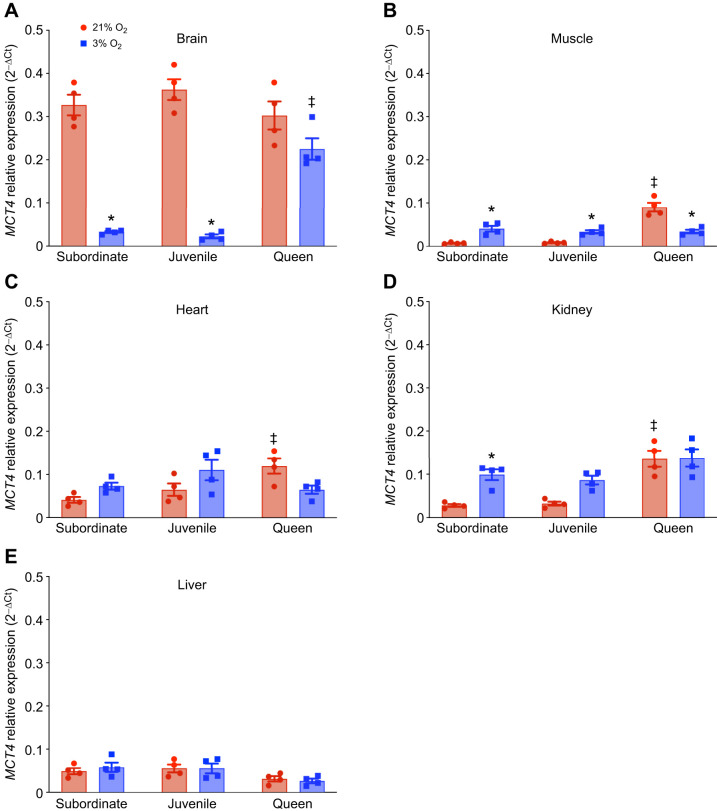
***Monocarboxylate transporter 4* (*MCT4*) gene expression across tissues and developmental stages in NMRs in normoxia and hypoxia (*n*=4 for each group).** Animals were exposed to 21% (red bars) or 3% O_2_ (blue bars) for 1 h. *MCT4* gene expression was measured during normoxia and hypoxia in (A) brain, (B) muscle, (C) heart, (D) kidney and (E) liver. Data are presented as means±s.e.m. Asterisks (*) indicate significant effects of hypoxia within each caste. Double daggers (‡) indicate significant differences between queens and subordinate animals (two-way ANOVA, *P*<0.05).

To determine which tissues might contribute to elevated blood lactate, we measured the expression of the *MCT4* gene across various tissues in all groups under normoxic and hypoxic conditions (*n*=4 for each tissue and treatment; Fig. 2; Table S1). MCT4 facilitates the export of lactate from cells, particularly in glycolytic tissues, preventing acid buildup during anaerobic metabolism (Halestrap and Price, 1999). *MCT4* expression is sensitive to hypoxia and is upregulated in cell lines during hypoxic conditions (Ullah et al., 2006). In normoxia, *MCT4* expression was highest in brain across all developmental stages (Fig. 2A) and was 2- to 3-fold lower in all other tissues (Fig. 2B–E). In addition, baseline *MCT4* expression was higher in queens than in juvenile or subordinate adults in normoxic muscle, kidney and heart tissues. With hypoxic exposure, *MCT4* expression decreased by ∼90% in subordinate and juvenile NMR brain, but was not significantly reduced in queens, remaining ∼5-fold higher than in other developmental stages treated with hypoxia. In muscle, heart and kidney, *MCT4* expression tended to increase with hypoxia in juvenile and adult subordinates, but this did not reach significance in all cases (Fig. 2B–D). In hypoxic queens, *MCT4* expression was decreased (muscle) or unchanged (heart and kidney). Liver *MCT4* expression was not impacted by hypoxia in any developmental stage (Fig. 2E).

### Divergent glycolytic enzyme changes across developmental stages during severe acute hypoxia

**Fig. 3.**
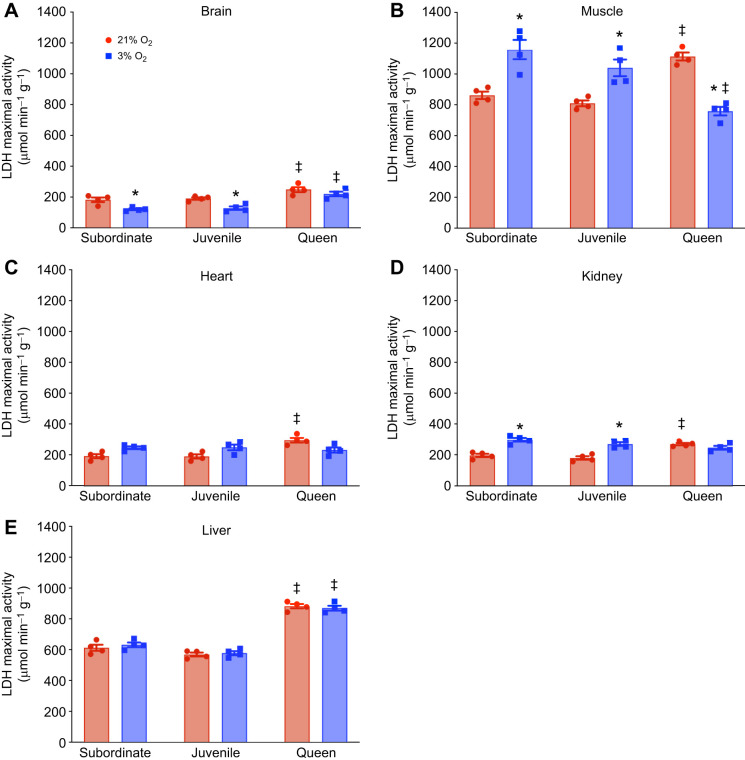
**Lactate dehydrogenase (LDH) enzyme activity across tissues and developmental stages in NMRs in normoxia and hypoxia (*n*=4 for each group).** Animals were exposed to 21% (red bars) or 3% O_2_ (blue bars) for 1 h. LDH activity per gram of tissue was measured during normoxia and hypoxia in (A) brain, (B) muscle, (C) heart, (D) kidney and (E) liver. Data are presented as means±s.e.m. Asterisks (*) indicate significant effects of hypoxia within each caste. Double daggers (‡) indicate significant differences between queens and subordinate animals (two-way ANOVA, *P*<0.05).

**Fig. 4.**
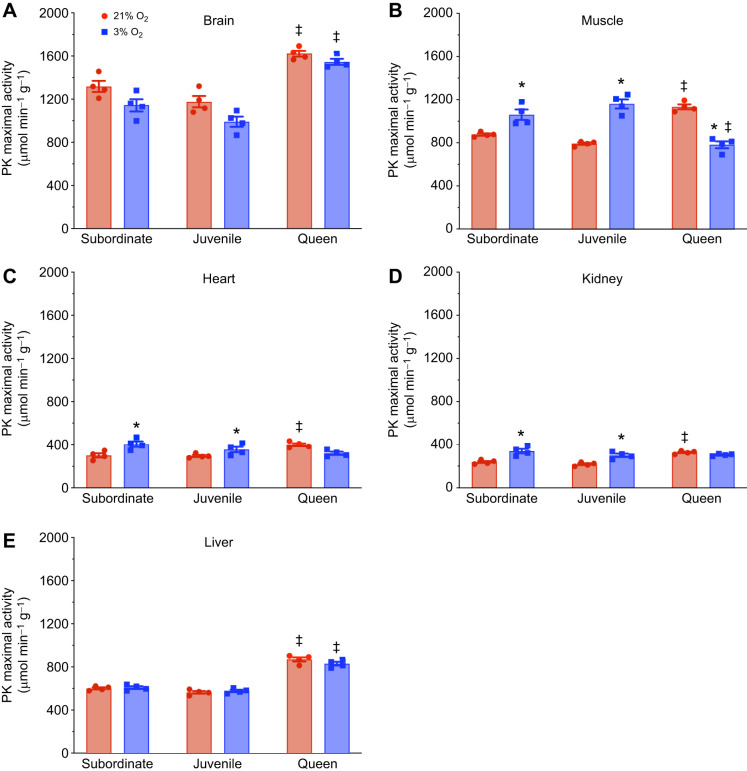
**Pyruvate kinase (PK) enzyme activity across tissues and developmental stages in NMRs in normoxia and hypoxia (*n*=4 for each group).** Animals were exposed to 21% (red bars) or 3% O_2_ (blue bars) for 1 h. PK activity per gram of tissue was measured during normoxia and hypoxia in (A) brain, (B) muscle, (C) heart, (D) kidney and (E) liver. Data are presented as means±s.e.m. Asterisks (*) indicate significant effects of hypoxia within each caste. Double daggers (‡) indicate significant differences between queens and subordinate animals (two-way ANOVA, *P*<0.05).

Next, we analyzed the anaerobic metabolic enzymatic activity of two enzymes that are central to anaerobic metabolism, LDH and PK. This analysis revealed distinct variations in activity across tissues of subordinate and juvenile animals compared with queens (*n*=4 for each tissue and treatment; Figs 3 and 4; Tables S2 and S3). In normoxia, baseline LDH and PK activity was high in muscle and liver and low in kidney and heart in all developmental stages. Interestingly, baseline LDH and PK activity in brain diverged, with PK activity being highest in brain in all developmental stages but LDH activity being lowest in brain in all developmental stages. Finally, normoxic LDH and PK enzyme activities were higher in queens than in subordinate adults or juveniles in all organs examined.

In hypoxia, enzyme activity tended to increase or be unchanged in non-breeding subordinate juveniles and adults but tended to decrease or be unchanged in queens. Specifically, LDH activity did not change following hypoxic exposure in the heart and liver of subordinates and juveniles, but increased by ∼30% in muscle and ∼50% in the kidney. Brain tissue was an outlier to this pattern, with LDH activity decreasing by ∼33% (Fig. 3). A different phenotype was observed in queens, in which there was no change in LDH activity in hypoxic brain, heart, liver or kidney, but a 32% decrease in muscle activity during hypoxia (Fig. 3). A similar pattern between developmental stage and enzyme activity was observed for PK. There were no changes in PK activity in the brain and liver of subordinates or juveniles. However, PK activity increased by ∼20% in the muscle of subordinates, ∼45% in the muscle of juveniles, ∼35% in the heart of subordinate adults and juveniles, and ∼40% in the kidney of both groups (Fig. 4). Conversely in queens, there was no hypoxia-mediated change in PK activity in the brain, heart, liver or kidney, but there was an ∼30% decrease in muscle (Fig. 4).

### Tissue-specific regulation of glycogen metabolism in NMRs under severe acute hypoxia

**Fig. 5.**
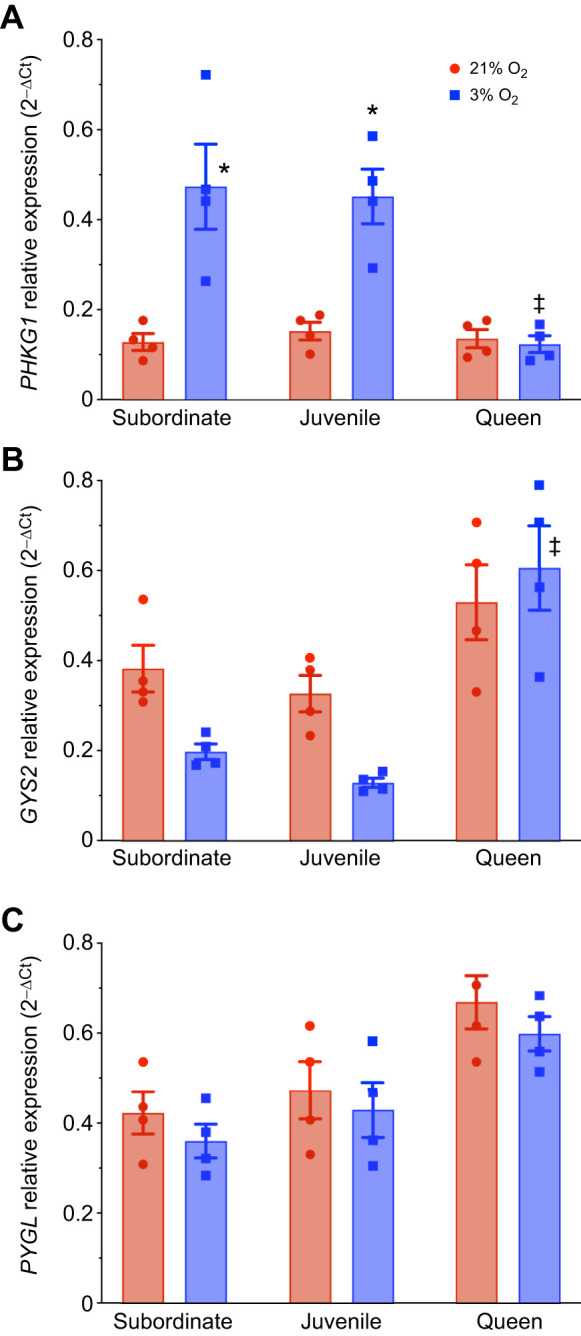
**The expression of liver glycogen enzymes is differentially regulated during hypoxia (*n*=4 for each group).** Animals were exposed to 21% (red bars) or 3% O_2_ (blue bars) for 1 h. Expression of the genes for phosphorylase kinase gamma 1 (*PHKG1*; A), liver glycogen synthase 2 (*GYS2*; B) and glycogen phosphorylase (*PYGL*; C) was measured across developmental stages in NMR liver. Data are presented as means±s.e.m. Asterisks (*) indicate significant effects of hypoxia within each caste. Double daggers (‡) indicate significant differences between queens and subordinate animals (two-way ANOVA, *P*<0.05).

**Fig. 6.**
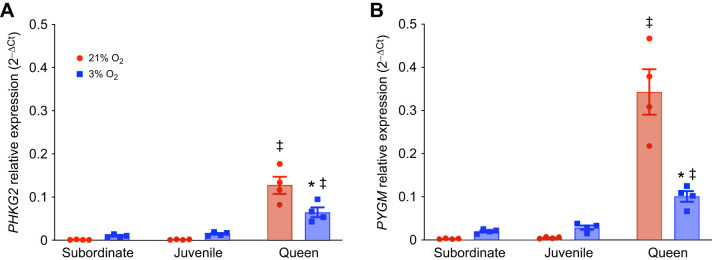
**The expression of muscle glycogen enzymes is differentially regulated during hypoxia (*n*=4 for each group).** Animals were exposed to 21% (red bars) or 3% O_2_ (blue bars) for 1 h. The expression of *phosphorylase kinase gamma 2* (*PHKG2*; A) and *muscle glycogen phosphorylase* (*PYGM*; B) genes was measured across developmental stages in NMR skeletal muscle. Data are presented as means±s.e.m. Asterisks (*) indicate significant effects of hypoxia within each caste. Double daggers (‡) indicate significant differences between queens and subordinate animals (two-way ANOVA, *P*<0.05).

**Fig. 7.**
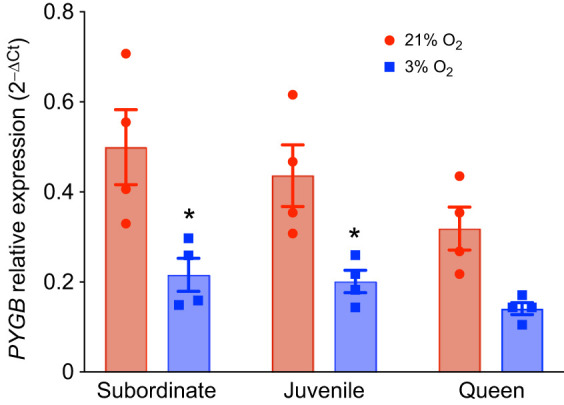
**The gene expression of *brain glycogen phosphorylase* (*PYGB*) decreases during hypoxia (*n*=4 for each group).** Animals were exposed to 21% (red bars) or 3% O_2_ (blue bars) for 1 h. Data are presented as means±s.e.m. Asterisks (*) indicate significant effects of hypoxia within each caste (two-way ANOVA, *P*<0.05).

Next, we measured the gene expression of enzymes involved in glycogen synthesis and glycogenolysis to understand how glycogen metabolism differs across developmental stages/castes and changes across different tissues under hypoxic conditions. Specifically, we examined *glycogen synthase 2* (*GYS2*) as an indicator of glycogen synthesis in liver; *phosphorylase kinase gamma 1* (*PHKG1*) and *glycogen phosphorylase* (*PYGL*) as indicators of glycogenolysis in liver; *glycogen phosphorylase* (*PYGM*) and *phosphorylase kinase gamma 2* (*PHKG2*) as indicators of glycogenolysis in muscle; and *glycogen phosphorylase* (*PYGB*) as an indicator of glycogenolysis in brain (*n*=4 for each tissue and treatment; Figs 5–7). This tissue-specific distinction is based on the well-documented roles of these isoforms in glycogen metabolism, as supported by previous studies (Agius, 2015; Roach et al., 2012).

In normoxic liver, the expression of all three genes was similar across developmental stages/castes, although *PYGL* was slightly higher in queens than in juvenile or subordinate adult animals (Fig. 5; Table S4). Conversely, with hypoxia exposure, the expression of *PHKG1* increased by ∼3-fold in the liver of subordinates and juveniles, but was unchanged in queens. *GYS2* and *PYGL* remained unchanged during hypoxia across all groups. Notably, *GYS2* expression decreased by ∼50% in both juveniles and adult subordinates, but this change did not reach statistical significance, likely owing to a low sample size.

In normoxic muscle, *PHKG2* and *PYGM* expression was almost undetectable in juvenile and adult subordinates but comparatively quite high in queens (Fig. 6; Table S4). With hypoxia exposure, the expression of *PHKG2* and *PYGM* was unchanged in juveniles and adult subordinates, whereas the expression of both enzyme genes decreased in queens (by ∼50% for *PHKG2* and by ∼70% for *PYGM*).

Next, we measured *PYGB* gene expression in brain. Note that we did not measure *PYGB* gene expression in other tissues because glucose is the main substrate for energy production in brain, whereas other tissues may rely on alternative substrates and metabolic pathways during hypoxia (Mergenthaler et al., 2013), which limits the conclusions that may be drawn in other tissues. We found that NMR brain *PYGB* gene expression was ∼50% lower during hypoxia across all developmental stages (*n*=4 for each group and treatment; Fig. 7; Table S4); however, this change was statistically significant only in the juvenile and subordinate groups.

### Opposing regulation of gluconeogenesis in queens versus subordinates and juveniles

**Fig. 8.**
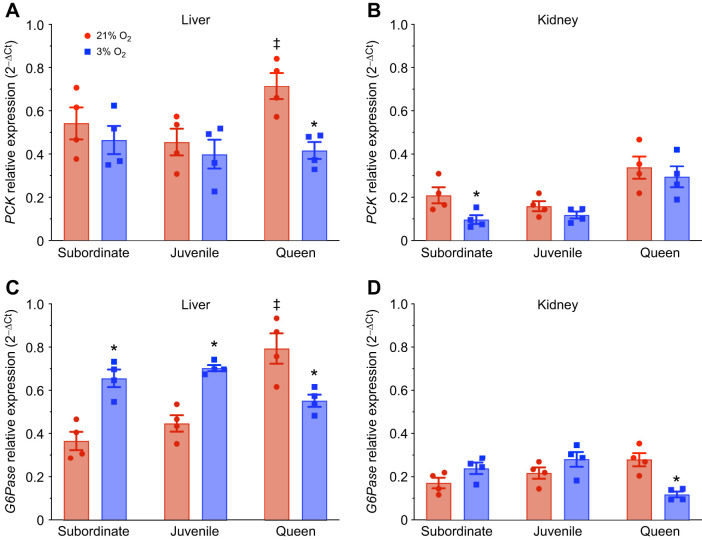
**The expression of gluconeogenic enzymes is differentially regulated during hypoxia (*n*=4 for each group).** Animals were exposed to 21% (red bars) or 3% O_2_ (blue bars) for 1 h. The expression of *phosphoenolpyruvate carboxykinase* (*PCK*) and *glucose-6-phosphatase* (*G6Pase*) genes was measured across developmental stages in NMRs: (A) *PCK* in liver, (B) *PCK* in kidney, (C) *G6Pase* in liver and (D) *G6Pase* in kidney. Data are presented as means±s.e.m. Asterisks (*) indicate significant effects of hypoxia within each caste. Double daggers (‡) indicate significant differences between queens and subordinate animals (two-way ANOVA, *P*<0.05).

Finally, we analyzed the expression of gluconeogenesis enzymes in the liver and kidney of subordinate, juvenile and queen NMRs under normoxic and hypoxic conditions to provide insight into whether this pathway is upregulated in NMRs, which might support the glycolysis pathway and prevent blood acidosis (*n*=4 for each tissue and treatment; Fig. 8). We found that the expression of both genes was higher in queen liver than in subordinate adult or juvenile liver, but no differences across castes were observed in kidney (Fig. 8; Table S5). Acute hypoxia increased *glucose-6-phosphatase* (*G6Pase*) expression in the liver by ∼80% in subordinates and 60% in juveniles, while it remained unchanged in the kidney. Additionally, the expression of *phosphoenolpyruvate carboxykinase* (*PCK*) remained unchanged in both tissues of juveniles and in the liver of subordinates, but it decreased in the kidney of subordinates. In contrast, the expression of *G6Pase* decreased in both tissues of queens during hypoxia (55% in the kidney and 30% in the liver). Finally, the expression of *PCK* decreased by ∼40% in the liver of queens but remained unchanged in the kidney during hypoxia.

## DISCUSSION

Our study provides several important advances in our understanding of how NMRs adapt to life in hypoxia, and how this is shaped by developmental stage and colony social structure. Specifically, our results provide insight into metabolic adjustments of NMRs to hypoxia and distinguishes divergent underlying regulatory strategies across developmental stages and/or hierarchical status within the colony in both systemic and tissue-specific contexts. First, we found that blood lactate levels increased during severe hypoxia across all developmental groups. In isolation, this finding suggests that all developmental stages of NMRs upregulate anaerobic metabolism at a systemic level in severe hypoxia (3% O_2_). However, our exploration of the underlying genes and enzymes involved in anaerobic metabolism reveal a divergent and tissue-specific phenotype across development and caste that supports our initial hypothesis, such that subordinate juveniles and adults have largely the same response to hypoxia, and queens employ a different strategy.

Specifically, subordinates upregulate *MCT4* expression and increase the activities of glycolytic enzymes in muscle and kidney, with a trend towards a similar response in heart tissue. Conversely, measurements in liver reveal no change in *MCT4* expression or glycolytic enzyme activity, suggesting no increase in anaerobic metabolism in this tissue. However, markers of glycogenolysis are upregulated, consistent with mobilization of liver glycogen stores to support blood glucose supply. We also report evidence of increased glucogenesis in liver with hypoxia. Together, these changes are expected to result in increased lactate export from cells to the blood, increased tissue (and particularly muscle) glycolytic activity, and mobilization of glucose from liver concomitant with elevated gluconeogenesis in the liver and kidney (Brooks, 2009; Choi et al., 2005; Hochachka and Somero, 2002; Powers and Jackson, 2008; Ullah et al., 2006). Intriguingly, an opposing phenotype was observed in brain, where hypoxia led to decreased mRNA expression of *MCT4*, and glycogenolysis and LDH enzyme activity. Taken together, and with the notable exception of brain tissue, these findings support increased reliance on anaerobic metabolism to meet energy demands and an ability to cycle glucose stores to sustain the supply of metabolic fuel and support carbohydrate metabolism in hypoxia.

This phenotype may reflect the roles and responsibilities of non-reproductive NMRs within the colony hierarchy. Specifically, subordinate juvenile and adult NMRs are very active within a burrow/colony setting, and regularly engage in intense aerobic work, including burrowing, tunnel maintenance, fighting off predators, and searching for and transporting food. Our findings suggest that juvenile and adult subordinates upregulate anaerobic metabolism in muscle, and to a lesser extent in kidney, to sustain activity-related energy demands associated with these tasks, even in hypoxia. Thus, the systemic increase in blood lactate in these developmental stages is likely due to increased anaerobic metabolism in exercising muscle.

In contrast, queens appear to have a greater reliance on anaerobic metabolism in normoxia but do not markedly upregulate systemic use of anaerobic metabolism in hypoxia. Specifically, and in sharp contrast to other developmental stages and castes examined, queens (1) reduce *MCT4* expression and glycolytic enzyme activity in muscle, (2) do not increase the expression of glycogenolysis enzymes in liver, but (3) do decrease the expression of glycogenolysis enzymes in muscle, and (4) downregulate the expression of glucogenesis-related enzymes in liver and kidney. In brain, *MCT4* expression is decreased, but this change is smaller than the reduction observed in juvenile and subordinate adults. In addition, the expression of glycogenolysis enzyme in the brain is maintained. Together, these findings suggest that queens prioritize the energetic demands of brain in hypoxia, but otherwise reduce their systemic reliance on anaerobic metabolism relative to their normoxic baseline. Thus, the systemic increase in blood lactate in queens may be the result of sustaining a relatively high level of brain function, supported in part by anaerobic metabolism, with the accumulated lactate circulating systemically until it can be oxidized following reoxygenation when environmental O_2_ levels are higher. This strategy may reflect the relatively sedentary lifestyle of queens within the nest of the NMR burrows, which may allow these animals to reduce physical activity (and thus skeletal muscle metabolism) and redirect energy to support the demands of hypoxia-sensitive brain.

### Temporal regulation of glycolysis in hypoxic NMRs

Intriguingly, previous studies of subordinate NMR muscle metabolism during longer hypoxia exposures indicate a reduction in glucose utilization, which contrasts with our findings in subordinates and juveniles treated with short-term acute hypoxia. Specifically, Hadj-Moussa et al. (2021) reported a downregulation of AMPK and its downstream effectors, including GLUT4, during longer-term severe hypoxia (3% O_2_ for 4 h). Similarly, Farhat et al. (2020) reported a decrease in glycolytic enzyme activity in muscle during chronic moderate hypoxia (11% O_2_ for 4–6 weeks). Together, these studies suggest that in certain conditions, NMR glucose metabolism may be reduced to conserve energy. However, our findings highlight that glucose metabolism is likely upregulated in subordinate juveniles and adults during severe acute hypoxia of 1 h, indicating that the glycolytic pathway is dynamically regulated to meet energy demands and the reliance on limited carbohydrate supplies may begin to decline with prolonged hypoxia exposure. These differences emphasize the complexity of the regulation of glucose metabolism in NMRs, where the balance between energy production and conservation is likely finely tuned based on the severity and duration of hypoxic stress.

### Reduced brain metabolism in hypoxia

An interesting finding in the present study pertains to the hypoxic brain, where markers of anaerobic metabolism generally trend toward downregulation in juveniles and subordinate adults. This pattern of a general reduction in glycolytic activity in brain aligns with previous findings of metabolic suppression in the brain of subordinate adult NMRs during hypoxia, in which global Na^+^/K^+^-ATPase activity and mitochondrial respiration are each reduced during acute or chronic *in vivo* hypoxia (Farhat et al., 2021a,b; Pamenter et al., 2018). The reduction of glycolytic activity, combined with a decrease in MCT4 expression, suggests that lactate production and export are minimized in the subordinate brain, likely as a strategy to prevent lactate accumulation and mitigate the risk of acidosis, which could impair neural function (Brooks, 2009; Schurr, 2006). Interestingly, acute hypoxia also decreases the expression of brain glycogen phosphorylase across all groups of NMRs. This finding supports our observations of reduced glycolytic enzyme activity and *MCT4* expression in the brain during hypoxia and aligns with previous studies demonstrating metabolic rate suppression in the brain under hypoxic conditions (Farhat et al., 2020, 2021b; Pamenter et al., 2018). Indeed, the expression of *MCT4* in normoxia is much greater in the brain than in other tissues (>6-fold), and its 90% reduction during hypoxia in subordinates and juveniles suggests that the brain might employ alternative pathways such as ketone utilization or amino acid metabolism to meet its energy demands and sustain neural activity under low O_2_ conditions in these developmental stages.

In addition, previous reports examining adaptations in the hypoxic brain of subordinate NMRs suggest a shift towards increased reliance on the pentose phosphate pathway, potentially as a protective mechanism to prioritize NADPH production for maintaining redox balance and defending against oxidative stress (Cheng et al., 2022; Eaton and Pamenter, 2022; Eaton et al., 2023). To support the pentose phosphate pathway, juvenile and subordinate NMRs may increase glucose-6-phosphate supply through enhanced glucose uptake, glycogenolysis, or possibly alternative pathways such as gluconeogenesis or lactate metabolism (Patra and Hay, 2014; Stincone et al., 2015).

Intriguingly, queens do not appear to modify brain MCT4 or glycolytic enzyme activity in hypoxia, in contrast to the strategy employed by subordinate animals. Given this divergent phenotype, the above experiments examining AMPK and ATPase activity in subordinate brains should be repeated in the brains of breeding NMRs to determine how metabolic signalling and metabolic demand differ in this key tissue in breeding animals.

### Maintained glycolytic activity in the hypoxic NMR heart

Our results indicate that glycolytic enzyme activity in the heart of subordinate, juvenile and queen NMRs remains unchanged except PK enzyme activity in subordinates, which increases. This finding contrasts with studies showing that NMRs possess enhanced glycogen stores in their cardiac muscle compared with hypoxia-intolerant mice, presumably to support a greater reliance on glucose utilization under low O_2_ conditions (Faulkes et al., 2019). Similarly, experiments on subordinate NMRs in chronic hypoxia (11% O_2_ for 4–6 weeks) demonstrate that although PK activity decreases, oxidation pathways are upregulated, allowing them to maintain their energy pathways in the heart to meet ATP demands, unlike other tissues where these pathways are suppressed (Farhat et al., 2020). These findings suggest that although NMRs possess large cardiac glycogen reserves, they may not increase cardiac glycolytic activity under severe and acute hypoxia, although they may be able to sustain anaerobic work for longer periods of time.

### Upregulation of genes involved in glycogenolysis in the liver and muscle, but not the brain

Acute hypoxia generally increases the expression of enzymes involved in glycogenolysis in the liver and muscle of juveniles and subordinate adult NMRs. Although results show that the expression of *PYGL* in the liver remains unchanged, the more pronounced increase in the expression of *PHKG1* suggests enhanced activation of glycogen phosphorylase through phosphorylation. These changes in the liver are expected to support elevated blood glucose levels during hypoxia (Pamenter et al., 2019), and presumably support anaerobic metabolism in vital organs such as the brain and heart. Conversely, as skeletal muscle lacks G6Pase, which is required for glucose release into the bloodstream (Nordlie et al., 1999; Roden and Bernroider, 2003), glycogenolysis in muscle serves as a local energy source rather than contributing to systemic glucose levels.

Interestingly, glycogenolysis enzymes in the muscle and liver of queens respond differently to hypoxia than in other groups. For example, the expression of *PYGM* in subordinates and juveniles is significantly lower than in queens under both normoxic and hypoxic conditions. These differences between groups, combined with the reduction in glycogenolysis enzymes in the muscle of queens, suggest that queens may prioritize energy conservation in muscle tissue while simultaneously leveraging carbohydrate metabolism. Furthermore, the expression of *PHKG1* and *PYGL* are unchanged in the liver of queens. These data indicate that the liver of queens continues to release glucose into the bloodstream during hypoxia like in normoxic conditions, presumably to support other tissues. Additionally, the lack of change in the expression of the GYS2 enzyme suggests that queens may be replenishing glycogen stores even under hypoxic conditions to maintain metabolic homeostasis.

### Severe acute hypoxia upregulates liver gluconeogenetic genes in subordinates and juveniles, but not queens

Previous studies demonstrate that gluconeogenic enzymes are expressed at significantly higher levels in the liver than the kidney, underscoring the predominant role of liver in glucose production (Gerich, 1993; van Schaftingen and Gerin, 2002). This is consistent with our findings, which support a central contribution of liver in gluconeogenesis. Specifically, acute hypoxia increases *G6Pase* expression in the liver of juvenile and subordinate adults, but its expression is reduced in hypoxic queens. G6Pase, the final enzyme in the gluconeogenesis pathway, is responsible for releasing glucose from glucose-6-phosphate (Hutton and O'Brien, 2009). In contrast, the expression of PCK, the initial enzyme in gluconeogenesis, is unchanged by hypoxia in the liver and kidney of juveniles and the liver of subordinates, but is reduced in the kidney of subordinates. The increased G6Pase activity in juveniles and subordinates may reflect a reliance on stored glycogen or non-glucose precursors such as glycerol that bypass the need for PCK (Hers and Hue, 1983; Pilkis and Granner, 1992). If this is the case, it would imbue metabolic flexibility that may help NMRs to optimize energy use during hypoxia by avoiding energetically costly pathways such as full gluconeogenesis while still maintaining some degree of glucose production to meet systemic demands. Notably, although the observed increase in G6Pase activity in the liver during hypoxia suggests enhanced glucose mobilization, likely to support systemic energy demands, our results do not directly confirm lactate utilization by the liver. Although lactate could potentially serve as a substrate for gluconeogenesis, this remains speculative in the absence of direct evidence. Future studies employing techniques such as isotopic tracing of lactate are necessary to determine whether lactate is actively taken up and utilized by the liver under hypoxic conditions. Nonetheless, it is clear that there is an increased reliance on anaerobic pathways in hypoxia to rapidly generate ATP from glucose in juvenile and adult subordinate NMRs. This may be linked to their ecological role within the colony, where they must sustain very energy-demanding behaviours in variable levels of hypoxia.

It is interesting to consider that we previously reported that subordinate NMRs do not experience metabolic acidosis in hypoxia (Pamenter et al., 2019), which is consistent with our current data, as subordinate NMRs increase gluconeogenesis, likely helping to reduce blood lactate levels. This strategy in subordinates and juveniles may help to ensure a continuous supply of glucose to meet energy demands in vital organs while conserving carbon molecules that would otherwise be lost through ventilation (Gladden, 2004). This would in turn reduce CO_2_ production by subordinates and juveniles during hypoxia, which may benefit queens by lowering the overall respiratory burden in crowded and poorly ventilated colony nest chambers and thus helping to stabilize blood pH levels for resident animals. This could also reduce the demand for pH buffering systems, thereby conserving energy (Hochachka and Somero, 2002; Robergs et al., 2004) and allowing queens to focus resources on reproduction and other vital functions during hypoxia.

### Study limitations

Although our study provides valuable insights into the metabolic responses of NMRs to hypoxia, several limitations should be acknowledged that may limit the conclusions that may be drawn from our results. First, although qPCR allows for the quantification of transcript levels, these do not always correlate directly with functional protein levels or enzymatic activity, particularly under non-steady-state conditions such as acute hypoxia. For instance, post-translational modifications, such as phosphorylation, which can modulate enzyme function independently of transcript expression, were not assessed in this study. Further study is warranted to explore potential roles for post-translational modification in these systems.

Additionally, although we measured plasma lactate levels, the lack of tissue-specific metabolite data, such as tissue-specific measurements of lactate, glycogen and glucose, limits our ability to draw conclusions about the metabolic contributions of individual tissues during hypoxia. Further experiments in this area would provide greater insight into the role of glycogenolysis, gluconeogenesis and lactate transport in specific organs. Furthermore, correlating the activities of key glycolytic enzymes, such as LDH and PK, with their respective transcript levels would strengthen the mechanistic interpretations of the observed changes and allow for a deeper exploration of metabolic adaptations to hypoxia across developmental stages and castes in NMRs.

### Conclusions

Our study provides a comprehensive analysis of tissue-specific metabolic responses to severe acute hypoxia across different developmental stages and castes in NMRs. Our findings indicate that acute hypoxia increases blood lactate levels in subordinate juveniles and adults as a result of enhanced glycolytic pathway activity, primarily in muscle. This likely supports the high glucose demands required for maintaining energy-intensive activities under hypoxic conditions. In contrast, although queens exhibit significantly higher blood lactate levels than other groups under normoxia, they appear to preferentially downregulate metabolic pathways outside of the brain during hypoxia. This suggests that queens prioritize energy conservation in hypoxia more so than subordinates, potentially to provide greater metabolic capacity to support nursing and reproduction. Unfortunately, we were unable to directly explore this question through analysis of tissues from pregnant or lactating queens because of the devastating impact their removal would have on a given colony.

Nonetheless, our results underscore divergent metabolic adaptations of NMRs in response to hypoxia, highlighting the importance of social rank and developmental stage in shaping strategies of hypoxia tolerance. The observed differences in metabolic responses between queens and non-reproductive animals may reflect the unique ecological and physiological pressures faced by each group. Importantly, these putative links between metabolism and behaviour are speculative; however, the distinct metabolic responses that we observed between castes align with the unique functional demands placed on each caste level and potentially demonstrate how physiological adjustments support colony survival under hypoxic conditions. Additional studies are warranted to explore whether the gene expression and enzyme activity differences described in the present study underlie functional differences in tissue and whole-animal metabolic responses to acute hypoxia across castes.

## Supplementary Material

10.1242/jexbio.250397_sup1 Supplementary information
