# Supplementary material for: Severe acute hypoxia upregulates anaerobic metabolism in non-reproductive but not queen naked mole-rats
Source: J Exp Biol. 2025 Jul 28;228(15):jeb250397. doi: 10.1242/jeb.250397 (PMC12377813; doi:10.1242/jeb.250397)
Supplement: Supplementary information [file jexbio-228-250397-s1.pdf]

Table S1. Statistical analysis of the effects of hypoxia and caste on MCT4 expression ($P < 0.05$).

Tissue	Effect of hypoxia	Effect of caste	Interaction
Brain	$F_{2,18}$ = 20.68, P < 0.0001	$F_{2,18}$ = 8.64, P = 0.0023	$F_{1,18}$ = 177.8, P < 0.0001
Heart	$F_{2,18}$ = 6.94, P = 0.0058	$F_{2,18}$ = 3.40, P = 0.0558	$F_{1,18}$ = 0.40, P = 0.5350
Liver	$F_{2,18}$ = 0.32, P = 0.7239	$F_{2,18}$ = 5.75, P = 0.0117	$F_{2,18}$ = 0.04, P = 0.8426
Muscle	$F_{2,18}$ = 40.96, P < 0.0001	$F_{2,18}$ = 35.48, P < 0.0001	$F_{1,18}$ = 0.01, P = 0.9114
Kidney	$F_{2,18}$ = 3.81, P = 0.0417	$F_{2,18}$ = 22.15, P < 0.0001	$F_{1,18}$ = 15.69, P = 0.0009

Table S2. Statistical analysis of the effects of hypoxia and caste on lactate dehydrogenase (LDH) enzyme activity ($P < 0.05$).

Tissue	Effect of hypoxia	Effect of caste	Interaction
Brain	$F_{2,18}$ = 1.154, P = 0.3377	$F_{2,18}$ = 26.94, P < 0.0001	$F_{1,18}$ = 24.99, P < 0.0001
Heart	$F_{2,18}$ = 11.30, P = 0.0007	$F_{2,18}$ = 6.138, P = 0.0093	$F_{1,18}$ = 1.792, P = 0.1973
Liver	$F_{2,18}$ = 0.55, P = 0.5853	$F_{2,18}$ = 223, P < 0.0001	$F_{1,18}$ = 0.18, P = 0.6759
Muscle	$F_{2,18}$ = 41.05, P < 0.0001	$F_{2,18}$ = 2.77, P = 0.0891	$F_{1,18}$ = 3.20, P = 0.0904
Kidney	$F_{2,18}$ = 19.05, P < 0.0001	$F_{2,18}$ = 4.43, P = 0.0271	$F_{1,18}$ = 37.70, P < 0.0001

Table S3. Statistical analysis of the effects of hypoxia and caste on pyruvate kinase (PK) enzyme activity ($P < 0.05$).

Tissue	Effect of hypoxia	Effect of caste	Interaction
Brain	$F_{2,18}$ = 0.88, P = 0.4297	$F_{2,18}$ = 61.82, P < 0.0001	$F_{1,18}$ = 15.16, P = 0.0011
Heart	$F_{2,18}$ = 13.04, P = 0.0003	$F_{2,18}$ = 1.83, P = 0.1876	$F_{1,18}$ = 3.43, P = 0.0841
Liver	$F_{2,18}$ = 2.1, P = 0.1514	$F_{2,18}$ = 222, P < 0.0001	$F_{1,18}$ = 0.31, P = 0.5821
Muscle	$F_{2,18}$ = 70.97, P < 0.0001	$F_{2,18}$ = 0.18, P = 0.8302	$F_{1,18}$ = 6.85, P = 0.0174
Kidney	$F_{2,18}$ = 14.54, P = 0.0002	$F_{2,18}$ = 10.74, P = 0.0009	$F_{1,18}$ = 28.77, P < 0.0001

Journal of Experimental Biology • Supplementary information

Table S4. Statistical analysis of the effects of hypoxia and caste on glycolytic enzyme expression ($P < 0.05$).

Tissue	Effect of hypoxia	Effect of caste	Interaction
Liver (PHKG1)	$F_{2,18} = 8.01$, $P = 0.0032$	$F_{2,18} = 8.33$, $P = 0.0027$	$F_{1,18} = 28.24$, $P < 0.0001$
Liver (PYGL)	$F_{2,18} = 0.03$, $P = 0.9679$	$F_{2,18} = 11.67$, $P = 0.0006$	$F_{1,18} = 1.86$, $P = 0.1891$
Liver (GYS2)	$F_{2,18} = 3.49$, $P = 0.0521$	$F_{2,18} = 19.35$, $P < 0.0001$	$F_{1,18} = 4.59$, $P = 0.0459$
Muscle (PHKG2)	$F_{2,18} = 10.38$, $P = 0.0010$	$F_{2,18} = 61.83$, $P < 0.0001$	$F_{1,18} = 3.24$, $P = 0.0886$
Muscle (PYGM)	$F_{2,18} = 23.22$, $P < 0.0001$	$F_{2,18} = 58.08$, $P < 0.0001$	$F_{1,18} = 13.62$, $P = 0.0017$
Brain (PYGB)	$F_{2,18} = 0.51$, $P = 0.6041$	$F_{2,18} = 3.197$, $P = 0.0649$	$F_{1,18} = 29.77$, $P < 0.0001$

Table S5. Statistical analysis of the effects of hypoxia and caste on gluconeogenic enzyme expression ($P < 0.05$).

Tissue	Effect of hypoxia	Effect of caste	Interaction
Liver (PCK)	$F_{2,18} = 2.33$, $P = 0.1254$	$F_{2,18} = 2.50$, $P = 0.1095$	$F_{1,18} = 8.064$, $P = 0.0109$
Kidney (PCK)	$F_{2,18} = 0.64$, $P = 0.5389$	$F_{2,18} = 15.30$, $P = 0.0001$	$F_{1,18} = 5.02$, $P = 0.0379$
Liver (G6pase)	$F_{2,18} = 24.79$, $P < 0.0001$	$F_{2,18} = 7.49$, $P = 0.0043$	$F_{1,18} = 8.46$, $P = 0.0093$
Kidney (G6pase)	$F_{2,18} = 11.76$, $P = 0.0005$	$F_{2,18} = 2.04$, $P = 0.1586$	$F_{1,18} = 0.18$, $P = 0.6692$

Journal of Experimental Biology • Supplementary information
